# Hepatitis C virus mediated chronic inflammation and tumorigenesis in the humanised immune system and liver mouse model

**DOI:** 10.1371/journal.pone.0184127

**Published:** 2017-09-08

**Authors:** Zhiqiang Zheng, Ching Wooen Sze, Choong Tat Keng, Muthafar Al-Haddawi, Min Liu, Sue Yee Tan, Hwee Ling Kwek, Zhisheng Her, Xue Ying Chan, Bhaskar Barnwal, Eva Loh, Kenneth Tou En Chang, Thiam Chye Tan, Yee-Joo Tan, Qingfeng Chen

**Affiliations:** 1 Institute of Molecular and Cell Biology, Singapore, Singapore; 2 Department of Microbiology and Immunology, Yong Loo Lin School of Medicine, National University Health System, National University of Singapore, Singapore, Singapore; 3 Department of Pathology and Laboratory Medicine, KK Women's and Children's Hospital, Singapore, Singapore; 4 Duke-NUS Graduate Medical School, Singapore, Singapore; 5 Department of Obstetrics & Gynaecology, KK Women's and Children's Hospital, Singapore, Singapore; 6 Key Laboratory for Major Obstetric Diseases of Guangdong Province, The Third Affiliated Hospital of Guangzhou Medical University, Guangzhou, China; Saint Louis University, UNITED STATES

## Abstract

Hepatitis C is a liver disease caused by infection of the Hepatitis C virus (HCV). Many individuals infected by the virus are unable to resolve the viral infection and develop chronic hepatitis, which can lead to formation of liver cirrhosis and cancer. To understand better how initial HCV infections progress to chronic liver diseases, we characterised the long term pathogenic effects of HCV infections with the use of a humanised mouse model (HIL mice) we have previously established. Although HCV RNA could be detected in infected mice up to 9 weeks post infection, HCV infected mice developed increased incidences of liver fibrosis, granulomatous inflammation and tumour formation in the form of hepatocellular adenomas or hepatocellular carcinomas by 28 weeks post infection compared to uninfected mice. We also demonstrated that chronic liver inflammation in HCV infected mice was mediated by the human immune system, particularly by monocytes/macrophages and T cells which exhibited exhaustion phenotypes. In conclusion, HIL mice can recapitulate some of the clinical symptoms such as chronic inflammation, immune cell exhaustion and tumorigenesis seen in HCV patients. Our findings also suggest that persistence of HCV-associated liver disease appear to require initial infections of HCV and immune responses but not long term HCV viraemia.

## Introduction

The hepatitis C virus (HCV) is a positive-strand RNA virus [1] that was estimated to currently infect 2–3% of the world’s population [2]. 50–80% of acute HCV infections progress to chronicity [3, 4] while the incidence of cirrhosis and hepatocellular carcinoma (HCC) in chronic HCV infections ranges from 15–35% and 1–3% respectively [5, 6]. Presence of HCV viraemia regardless of viral titres or genotype is a major risk factor for the development of HCC [6–8]. One of the milestones in HCV research is the recent discovery of direct acting antivirals against HCV which, when used in appropriate combinations is effective against various genotypes of HCV in infected individuals [9–13]. Although patients who achieve sustained virologic response (SVR) have a substantially reduced risk of HCC [14] and it is hopeful that we will soon be able to achieve SVR in most HCV infected patients, the precise mechanisms of HCV pathogenesis are still not well understood. Furthermore, a proportion of patients who achieved SVR still develop HCC [15–17], hence, a better understanding of HCV pathogenesis is required for the development of therapeutic strategies to manage viral mediated tumorigenesis.

One of the main obstacles for studying HCV pathogenesis is the restriction of HCV tropism in humans. Currently, chimpanzees represent the most relevant animal model that can support HCV infection and recapitulate host responses and clinical symptoms similar to those observed in human patients. However, disadvantages of the primate model such as high cost, poor chronic infection rates and ethical concerns have limited their applicability in hepatitis research. To address these issues, small animal models that can accommodate HCV infections have been developed. These include transgenic mice strains modified to express factors to allow HCV permissiveness in mouse hepatocytes [18]. Genetically modified strains that allow efficient chimaerisms of mouse livers with transplanted human hepatocytes such as the SCID/Alb-uPA [19], Fah/Rag2/Il2rg [20], AFC8-hu HSC/Hep [21] and TK-NOG [22, 23] mice have been shown to support HCV infections. We recently described the Humanised Immune system and Liver (HIL) mice which supports chimaerism of both the immune component and livers with cells from the same foetal liver donor. This can be achieved by introducing CD34+ purified human foetal liver cells, which contains a proportion of CD34^hi^CD133^hi^ haematopoietic progenitors and CD34^lo^CD133^lo^ hepatic progenitor cells into immunodeficient NOD-scid Il2rg^-/-^ (NSG) mice [24]. Unlike the other humanised mouse models, HIL mice do not require additional transgene modifications or drug treatments to induce cell death of mouse hepatocytes. In a recent study, we described successful infections of HIL mice with HCV, resulting in the development of HCV-specific human immune responses and clinical symptoms such as liver inflammation and fibrosis by 9 weeks post infection, which, could be ameliorated by treatment with interferon alpha-2a [25].

In this report, we investigated further, the long term effects of hepatitis C pathogenesis in HIL mice up to 28 weeks post infection. Although long term viraemia was not detected in these mice, HCV infected HIL mice developed increased incidences of liver inflammation, fibrosis, steatosis and rare occurrences of liver tumours. Analyses of the immune profiles of HCV infected mice showed an expansion of monocytes/macrophages, particularly of the pro-tumorigenic M2 phenotype and T cells with increased numbers of exhaustion-like CD4 T helper populations. Our results suggest that an initial infection of HCV is sufficient to drive chronic hepatitis which may subsequently promote liver tumorigenesis.

## Materials and methods

### Ethics statement

For the construction of humanised mice, human foetal livers were obtained from elective or medically indicated termination of pregnancies after obtaining informed and written consent from the donors through a non-profit collaboration with the KK Women’s and Children’s hospital. Identities of the patients were not provided or traceable. The collection of donor tissue for constructing humanised mice for the purpose of studying human infectious diseases was reviewed and approved by the SingHealth centralised institutional review board (CIRB Ref: 2012/064/F). Samples from 3 different donors were used to construct the HIL mice used in this study (S1 Table).

All animal experiments were conducted according to protocols approved by our institution’s (Biological Resource Centre, Agency of Science, Technology and Research, Singapore) institutional animal care and use committee (IACUC). Approval of IACUC protocols for the construction of humanised mice (IACUC #120716 and #151034) and the establishment of a mouse model for hepatitis C virus infection suitable for studying viral-host interactions in vivo (IACUC #120740 and #151037) were obtained for the course of this study.

### HIL mice

NSG mice were purchased from the Jackson Laboratory, bred and housed under specific pathogen free (SPF) conditions in animal facilities at the Agency of Science, Technology and Research’s Biological Resource Centre in Singapore. The construction and infection of HIL mice with HCV were performed as previously described [25]. Briefly, one to three day old NSG pups were irradiated with a sub-lethal dose of 1Gy and transplanted with 2x10^5^ CD34+ human foetal liver cells via intra-hepatic injections. Mice were intravenously infected with HCV 8–12 weeks post-transplantation and studied for a period of 27–28 weeks. A total of 44 HIL mice were used in this study (S1 Table). Animal health status, water and food uptake were monitored daily. Animals were euthanised by carbon dioxide asphyxiation at experimental endpoints or if there was evidence of weight loss of more than 10% body weight within 24 hours or more than 20% of their original weight prior to infections or major signs of animal distress such as hunched posture, weakness or inability to feed or drink. All efforts were made to minimise animal suffering.

### HCV production and titration

A laboratory HCV strain known as the J6/JFH1-P47 [26] was used throughout this study. Production and viral titration were performed as previously described [27].

### Analysis of human serum albumin or serum alanine aminotransferase levels

When indicated, 50–100 μl of blood was collected into EDTA-treated tubes by bleeding the facial veins of mice. Facial vein bleeding was carried out without the use of anaesthetics or analgesics as this procedure is minimally invasive and is an established method of sampling blood from conscious mice. Blood samples were centrifuged at 2000 x g for 10 minutes at 4°C and the plasma fraction was collected for further analysis. Human serum albumin (HSA) levels were determined using the human albumin ELISA kit (Bethyl Laboratories) according to the manufacturer’s instructions. Serum alanine aminotransferase levels were measured using a Cobas C111 analyser (Roche) in the veterinary diagnostic laboratory at the National University of Singapore.

### Immunohistochemical and immunofluorescence staining

Mouse livers were collected at the specified time points, fixed with 10% neutral buffered formalin and embedded in paraffin for processing into 4 micron sections. Sections were rehydrated and stained with Haematoxylin and Eosin (H&E) (Thermo Scientific) or Sirius red and Fast green (Sigma) before pathology evaluation. Alternatively, sections were treated with heat mediated antigen retrieval with pH6 sodium citrate or pH9 Tris-EDTA buffer before staining with primary antibodies. Primary antibodies used in this study include anti-HSA (ab2406, ab19180 Abcam), anti-CD3 (ab699, Abcam), anti-CD20 (555677, BD), anti-CD68 (ab955, Abcam), anti-PD1 (ab137132, Abcam), anti-β-catenin (610154, BD), anti-glutamine synthetase (MAB302, Millipore), anti-LFABP (ab190958, Abcam), anti-IL6 (ab6672, Abcam) and anti-IL10 (ab34843, Abcam).

Immunohistochemistry staining was performed using the Superpicture 3^rd^ Gen IHC Detection Kit (879673, Life Technologies) while immunofluorescence staining was done using anti-mouse IgG, anti-rabbit IgG or anti-Goat IgG secondary antibodies conjugated to Alexafluors 488 or 647 (Life Technologies). Images were captured using an Olympus upright confocal microscope or a Zeiss Axioscan Z1 slide scanner.

### HCV Fluorescent RNA in situ hybridisation

Formalin fixed liver sections were prepared as per immunohistochemical staining and stained using the Quantigene ViewRNA ISH tissue assay kit (QVT0012, Affymetrix) and co-stained with anti-HSA (ab2406, ab19180 Abcam) antibodies according to the manufacturer’s instructions. Two type 6 probe sets specific for HCV RNA (VF6-18614, VF6-11508) were used in this study. Images were captured using an Olympus upright confocal microscope.

### Pathological analyses of liver abnormalities

Liver sections were examined as blinded studies by a pathologist at the advanced molecular pathology unit (AMPL) at the institute of molecular and cell biology in Singapore. Key observations made included liver fibrosis, necrosis, steatosis, granulomatous inflammation, focal nodular hyperplasia and the presence of liver tumours in the form of hepatocellular adenomas or hepatocellular carcinomas.

### Flow cytometry

Mononuclear cells were extracted from livers and spleens as previously described [24]. Antibodies against human CD3 (564307, BD), CD4 (563550, BD), CD14 (325608, BD), CD19 (563325, BD), CD45 (562279, BD), CD45RA (304106, BD), CD56 (304606, BD), CD69 (563835, BD), CD152/CTLA4 (349914, Biolegend), CD197/CCR7 (563449, BD), CD279/PD1 (329928, Biolegend), HLADR (641393, BD) and mouse CD45.1 (110730, BD) were used for the staining of leukocytes. Flow cytometry data was acquired using a BD LSRII flow cytometer and analysed with the Flowjo software (Treestar).

### Cytokine detection

Detection of inflammatory cytokines, IFN-γ, MCP-1, IL6, IL8, IL10, IL12p70, IL17A, IL18 and IL23 from plasma samples were performed using the Legendplex multi-analyte flow assay kit (Biolegend) according to the manufacturer’s instructions.

### Statistical analyses

Statistical analyses were done using a two-tailed Student’s T test to evaluate the statistical significance of data generated. Differences in measured data were considered to be statistically significant when p < 0.05.

## Results

### HCV infected HIL mice have elevated serum ALT and HSA levels

To address the potential variations due to donor genetics, three cohorts of mice humanised from different donors were studied (S1 Table). For cohort 1, mice were mock infected (PBS, Huh7.5 culture media) or infected with 10^6^ or 10^7^ ffu of HCV. For cohorts 2 and 3, mice were mock infected or infected with 10^7^ ffu of HCV. At eight weeks post reconstitution, HIL mice used in this study had a mean of ~39% human immune cell reconstitution in peripheral blood and 23.79 ng/mL of HSA. To study the long term effect of HCV infection, HIL mice were intravenously injected with mock, 10^6^ or 10^7^ ffu of J6/JFH1-P47 HCV (Genotype 2a) between eight to twelve weeks after reconstitution and followed up for twenty eight weeks.

**Fig 1:**
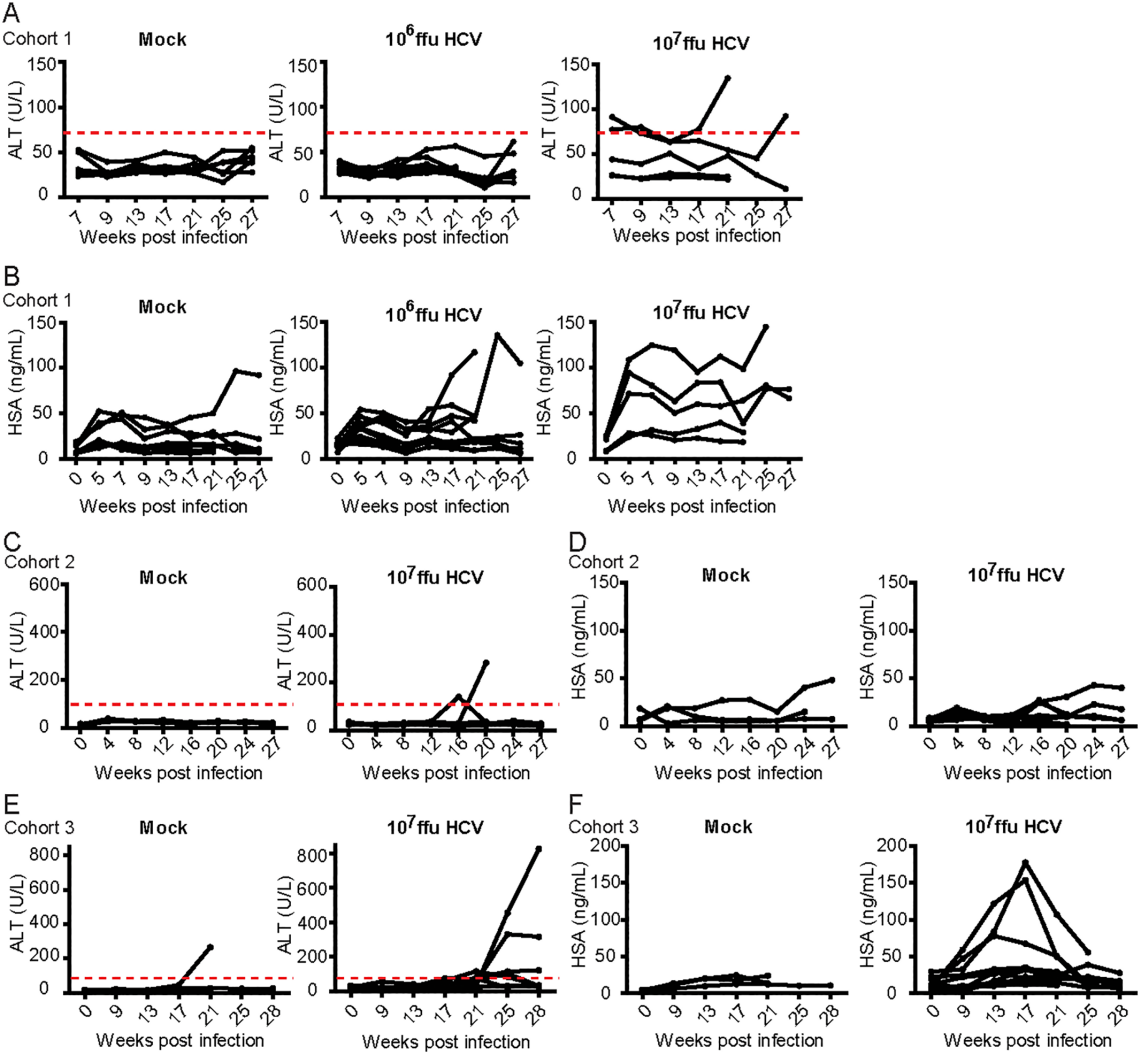
Longitudinal serum ALT and human albumin levels in HIL mice post HCV infection. 3 cohorts of HIL mice were either mock infected or infected with a 10^6^ or 10^7^ ffu dose of HCV. For mice that survived more than 8 weeks, serum taken from each mouse every month were analysed (Cohort 1: mock n = 8, 10^6^ ffu HCV n = 10, 10^7^ ffu HCV n = 10. Cohort 2: mock n = 10, 10^7^ ffu HCV n = 13. Cohort 3: mock n = 3, 10^7^ ffu HCV n = 11). (A, C, E) Serum ALT and (B, D, F) HSA levels were measured at various time points for a period of 27–28 weeks post HCV infection.

Serum alanine aminotransferase (ALT) levels were measured prior to and at monthly time points after the introduction of HCV as a non-invasive method for assessing the presence of liver damage (Fig 1). Normal ALT values published for mice are 17–77 U/L. In the first cohort of mice (S1 Table), Mock infected mice had ALT levels ranging from 18.64 to 53.12 U/L. None of the mice infected with 10^6^ ffu dose of HCV had increased ALT levels whereas 2 of 5 (40%) of mice infected with a 10^7^ ffu dose of HCV had elevated serum ALT levels (Fig 1A). Based on this observation we surmised that a higher dose of HCV induced a higher incidence of liver damage. Subsequent cohorts of mice were therefore infected with a 10^7^ ffu dose of HCV. For cohorts 2 and 3, mock infected mice had ALT levels ranging from 6.08 to 39.36 U/L (cohort 2) and 12.16 to 265.92 U/L (cohort 3) throughout the course of study. 1 of 6 (17%) mouse from the mock infected group and 7 of 16 (44%) mice from the group infected with a 10^7^ ffu dose of HCV had elevated serum ALT levels (Fig 1C and 1E).

HSA levels were also measured to gauge the initial level of human hepatocyte reconstitution and to track the subsequent proliferation of the human hepatocytes during the course of the study. With the exception of 1 mouse, levels of HSA appeared constantly under 50 ng/mL in 12 of 13 (92%) mock infected mice while 3 of 10 (30%) and 6 of 21 (29%) mice infected with a 10^6^ or 10^7^ ffu dose of HCV respectively, showed elevated levels of HSA ranging from 54 to 180 ng/mL (Fig 1B, 1D and 1F).

In cohorts 1, the earliest elevations of serum ALT levels were observed at 7–16 weeks post HCV infection. However, increased serum ALT levels were only recorded after 16–21 weeks post HCV infection in cohorts 2 and 3, which could be attributed to the difference of human donor genetics. Elevations of HSA were constantly observed by 9 weeks post infection in all 3 cohorts, although no obvious correlations between increased serum ALT and HSA levels were noted (S2 Table). These results suggest that human hepatocytes in HIL mice may undergo expansion after HCV infection. Readings of serum ALT and HSA are also available as (S3 Table).

### HCV infected HIL mice do not develop long term viraemia

We previously reported that HCV RNA could only be detected after purification of human epidermal growth factor receptor expressing cells from mouse livers but not by directly assaying total RNA extracted from mouse plasma or livers [25]. To increase the sensitivity of our HCV detection assay, a pre-amplification step was performed prior to qPCR. Plasma from HIL mice spiked with known amounts HCV RNA were used to determine the sensitivity of this assay to a detection limit of 23 copies of HCV RNA per 100 μl of plasma. However, no HCV RNA could be detected from plasmas of infected HIL mice at 5, 9 or 28 weeks post infection (Panel A in S1 Fig). Similarly, although we were able to detect HCV RNA in fixed liver sections in mice 9 weeks post HCV infection in an RNA in situ hybridisation assay, no HCV RNA could be detected from any HCV infected mice at 20–28 weeks post infection (Panel B in S1 Fig). Collectively, these findings suggest that there was no detectable long term viraemia in these mice.

### HCV infected HIL mice developed liver pathological changes

**Fig 2:**
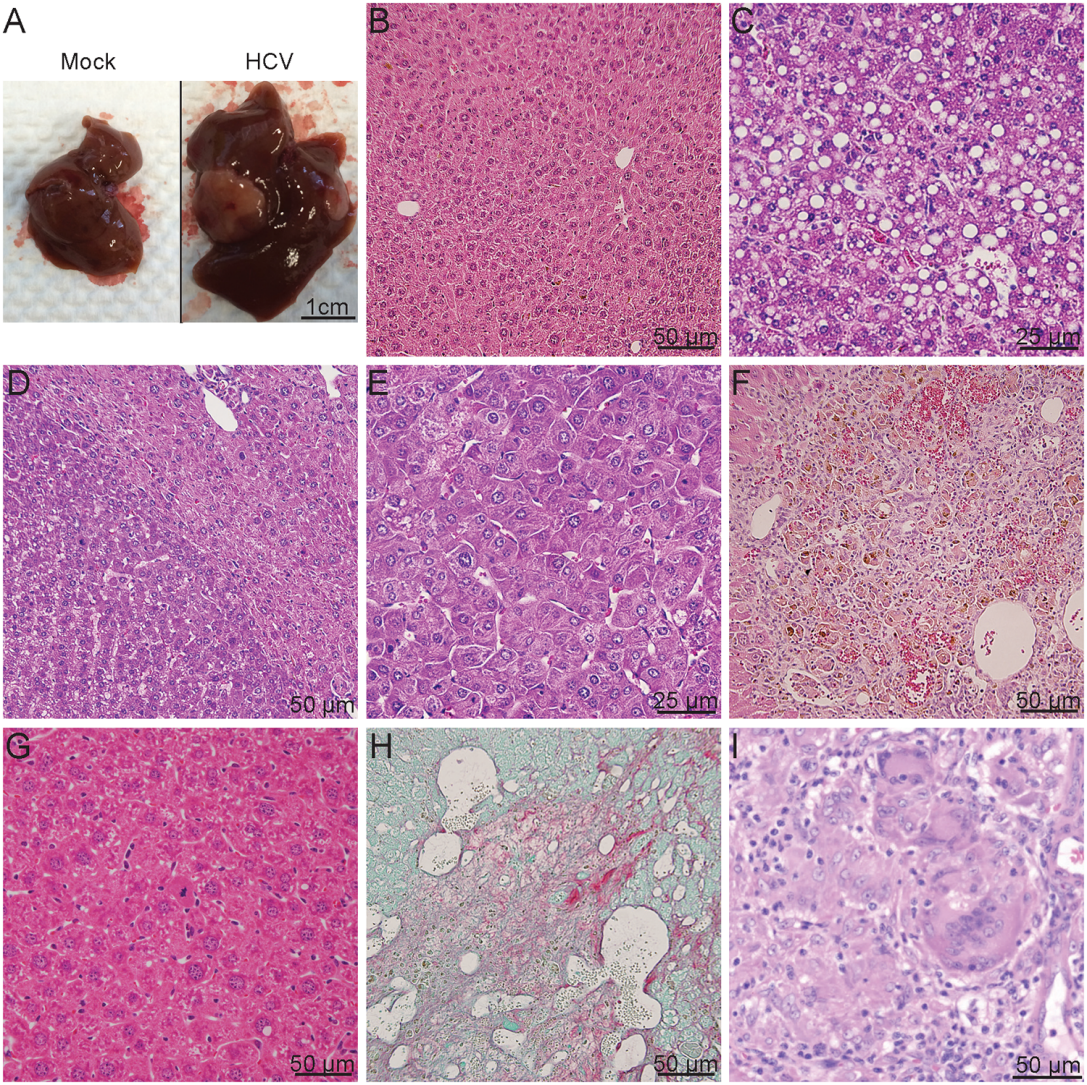
Liver abnormalities detected in HCV infected mice at 27 weeks post infection. (A-H) Livers were harvested from mice in the mock and HCV infected groups, paraffin-embedded and processed for staining with (B-G) haematoxylin and eosin or (H) Sirius red/fast green stains. (A) Representative image showing gross appearance of livers obtained from either mock or HCV infected mice at 27–28 weeks post infection. Representative image of H&E stains showing (B) normal liver from mock infected mice and (C) steatosis, (D) hepatocellular adenoma, (E) hepatocellular carcinoma, (F) granulomatous inflammation, (G) focal nodular hyperplasia and (H) liver fibrosis from livers harvested from HCV infected mice.

Although monitoring of serum ALT levels is one of the most commonly used non-invasive diagnostic methods for detecting liver damage, many HCV infected patients with significant liver disease present normal levels of serum ALT [28, 29]. To confirm the presence of liver disease, histological analyses were performed on livers harvested from HIL mice between 20 to 28 weeks post infection from three different cohorts of mice (Fig 2 and Table 1). Mice that did not survive beyond 20 weeks post HCV infection were excluded from this study. Livers harvested from mock infected mice appeared normal (Fig 2A) which was confirmed by H&E staining (Fig 2B) while superficial lesions of nodular patterns were observed in some of the livers from HCV infected mice (Fig 2A). A total of 10 mice were infected with a 10^6^ ffu dose of HCV. None from this group were found to have developed liver fibrosis, which could be visualised by Sirius Red/Fast green stains, however, 3 (30%) were found with liver necrosis, 1 (10%) with focal or diffuse hepatic vacuolation, indicative of liver steatosis and 8 (80%) with granulomatous inflammation. No benign or malignant neoplasms were detected in this treatment group at 20–28 weeks post infection (Table 1).

**Table 1:** Frequencies of liver abnormalities in HCV infected mice.

	Fibrosis	Necrosis	Steatosis	Granulomatous inflammation	Focal nodular hyperplasia	Hepatocellular adenoma	Hepatocellular carcinoma
Mock	0/13	4/13	0/13	4/13	0/13	0/13	0/13
10^6^ HCV	0/10	3/10	1/10	8/10	0/10	0/10	0/10
10^7^ HCV	5/21	7/21	1/21	16/21	1/21	2/21	1/21
Total HCV	5/31	10/31	2/31	24/31	1/31	2/31	1/31

Of the 21 mice infected with 10^7^ ffu HCV, 1 (5%) was found with steatosis (Fig 2C), 7 (33%) with liver necrosis, 2 (10%) with liver tumours in the form of hepatocellular adenomas (Fig 2D) and carcinoma (Fig 2E), 16 (76%) with granulomatous inflammation (Fig 2F), 1 (5%) with focal nodular hyperplasia (Fig 2G) and 5 (24%) were found to have liver fibrosis (Fig 2H). Compared to the group of mock infected mice, both HCV infected groups had increased frequencies of liver fibrosis, steatosis and granulomatous inflammation. Developments of liver necrosis were seen in around 20–30% of all mice from both infected and mock infected groups, suggesting that necrosis was not caused by HCV infection but by other factors such as the old age of mice at the time of analysis. No signs of liver fibrosis, steatosis or tumours were detected in mock infected mice, although 2 (20%) of 10 mice had minimal liver necrosis and 4 (31%) were found to have minimal granulomatous inflammation (Fig 2 and Table 1).

### Liver tumour formation in HCV infected HIL mice

As shown above, we observed the formation of both hepatocellular adenomas and carcinomas in 10% of HCV infected mice and 5% with benign growths in the form of focal nodular hyperplasia (Table 1 and Fig 2). Intranuclear cytoplasmic invaginations of acidophilic inclusions were also observed within the adenomas (Fig 2D).

**Fig 3:**
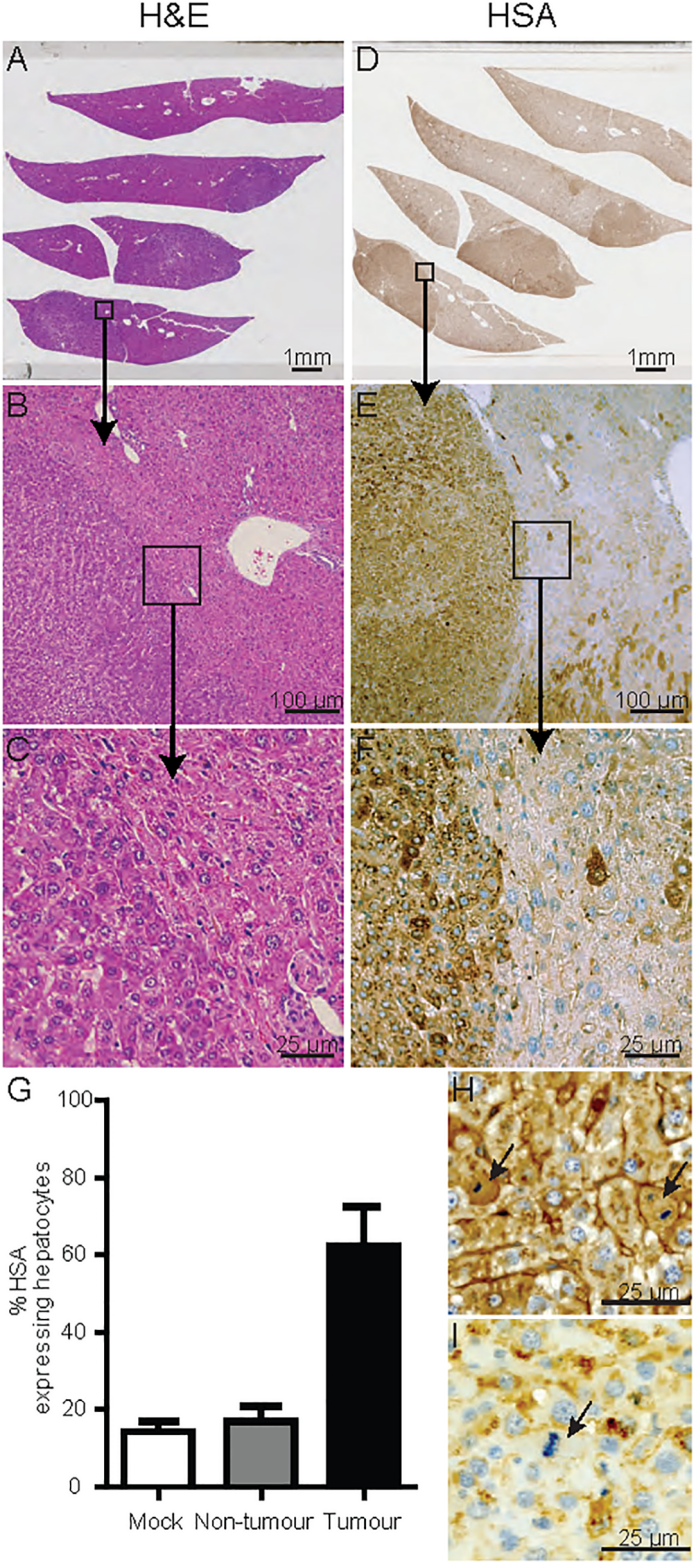
Majority of dividing cells in liver tumours are of human origin. (A-C) H&E stain of a hepatocellular adenoma containing liver section. (B&C) Nodular growth, loss of normal architecture with irregular growth pattern and compression of the surrounding parenchyma. (D-F) Serial section stained using an antibody specific against HSA showing 80–90% human albumin positivity within the liver tumours. (G) HSA expressing hepatocytes of mock (n = 3), non-tumour (n = 2) and tumour (n = 2) regions within liver sections were counted and expressed as a percentage of total hepatocytes. Percentages represent averaged counts of HSA expressing hepatocytes against total hepatocytes from five randomly selected 500 x 250 μm regions per sample. Error bars represent standard error of the means. (H-I) Representative images of mitotically active hepatocytes that (H) express HSA or (I) do not express HSA.

The presence of multiple tumour types were noted in some mice, for instance, two separate regions of hepatocellular carcinoma were detected in the liver section from one HCV infected mouse (Fig 3A). Both regions of hepatocellular carcinoma localised within larger hepatocellular adenomas (Fig 3B and 3C), suggestive of malignant transitions of the hepatocellular adenomas into hepatocellular carcinomas. To assess if the HCC containing tumour regions were populated by cells of human or mouse origin, liver tumour sections were stained for human serum albumin. Results revealed that the tumours were comprised of a mixture of both human and mouse cells (Fig 3D–3F). Other liver tumours formed in this study were also analysed using the same methodology, revealing similar results, suggesting that all tumours contained both human and mouse cells. Percentages of human albumin^+^ cells vary between tumours, ranging from an estimated 33% to 91% with a mean of 62% whereas adjacent non-tumour regions range from 16 to 18% with a mean of 17%. Mock infected mice show similar percentages of HSA positive cells to non-tumour regions with a range of 9 to 18% with a mean of 14% (Fig 3G). These results suggest that in HCV infected HIL mice, human hepatocytes were specifically transformed into tumour cells and mouse cells in the same vicinity of actively dividing human tumour cells were engulfed and incorporated into the tumours. This hypothesis was further evidenced by pathological analyses: 29 randomly sampled mitotic cells within various tumours were counted, of which 27 expressed HSA (Fig 3H) whereas 2 did not (Fig 3I), demonstrating that a majority (93%) of actively dividing cells within the liver tumours were human cells. To exclude the possibility of non-specific binding of the anti-HSA antibody, liver sections from wild-type mice and mice with CpG oligodeoxynucleotide induced liver tumours were used as controls for staining. No positive staining was observed in the mouse hepatocytes in normal mouse livers (Panels A and B in S2 Fig) or mouse tumours, although regions of mouse tumours showed higher background compared to normal livers (Panels C and D in S2 Fig). These results confirmed that the staining in HIL mouse livers and tumours were specific for HSA (Panels E and F in S2 Fig).

The hepatocellular adenomas found in HCV infected HIL mice were further characterized by the expression of β-catenin, glutamine synthetase and liver fatty acid binding protein (LFABP) according to previous studies [30–32]. Liver sections from both mice with hepatocellular adenomas showed no nuclear localisation of β-catenin, with negative or focal staining of glutamine synthetase suggesting that none of the adenomas were β-catenin mutated (Panels A and B in S3 Fig). LFABP staining was negative in all adenomas from one HCV infected mouse (Panel A in S3 Fig) and positive in all adenomas from the other HCV infected mouse (Panel B in S3 Fig). Although only two HCV infected HIL mice in this study developed hepatocellular adenomas, our data demonstrates that both HNF1a inactivated and inflammatory adenomas can develop in this model.

### Human CD3 T cells and CD68 monocytes/macrophages are the major immune cell types present in chronically inflamed HCV infected livers

**Fig 4:**
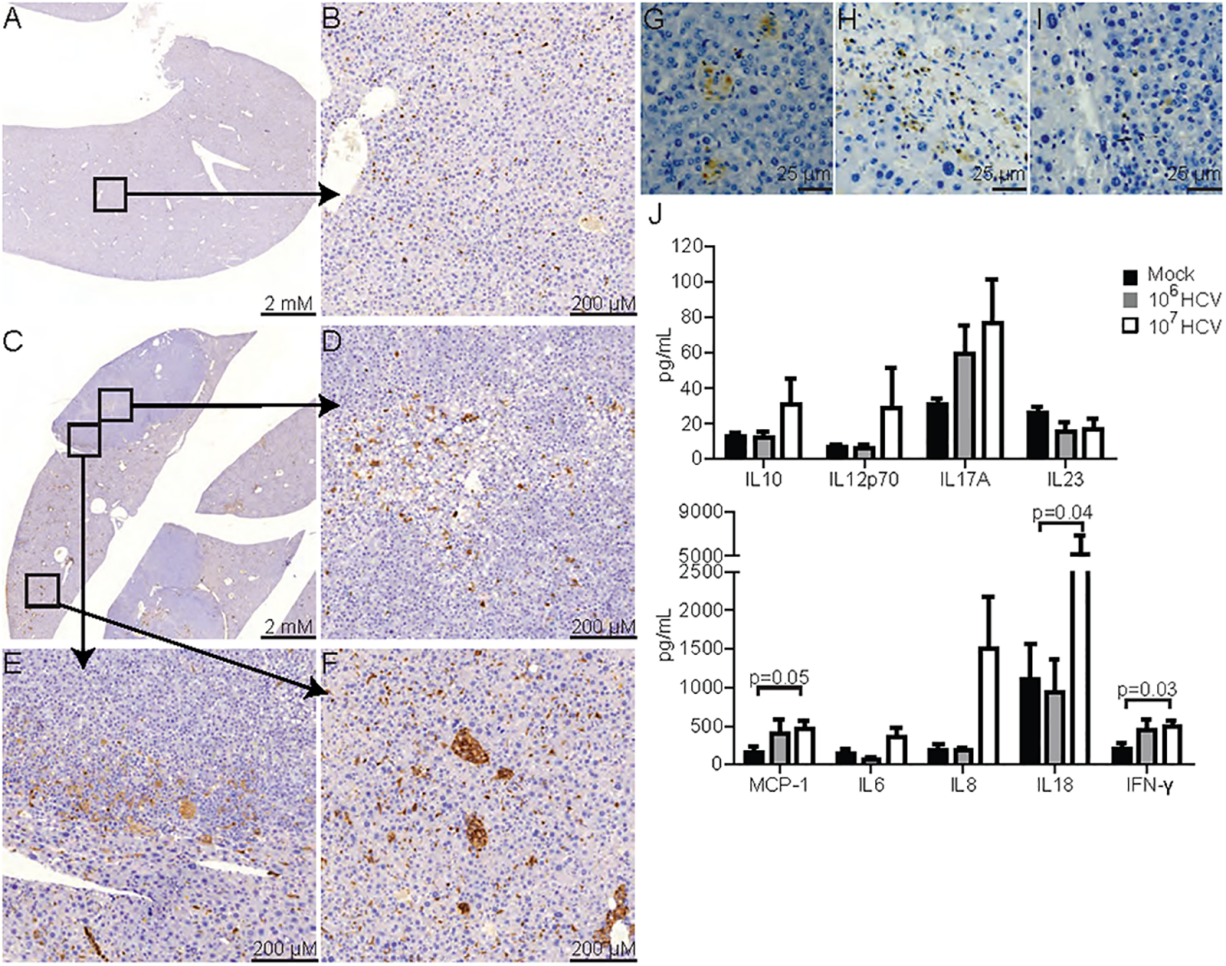
Human immune cell infiltration and inflammation in HCV infected HIL mice. Liver sections from (A-B) mock infected or (C-F) HCV infected HIL mice 27 weeks post infection were stained for human CD45 and counterstained with haematoxylin. Representative images showing (A) liver from mock infected HIL mice, (B) high magnification of an area in mock infected mice (Squared region), (C) liver from HCV infected HIL mice, (D-E) high magnification of areas in a hepatocellular adenoma from HCV infected mice, and (F) high magnification of an area in a non-tumour region from HCV infected mice. (G-I) Representative images showing human (G) CD68, (H) CD3 and (I) CD20 stains within hepatocellular adenoma in HCV infected mice. (J-K) Plasma samples at 27–28 weeks post infection were quantified for the presence of human cytokines: (J) IL10, IL12p70, IL17A, IL23 (K) MCP-1, IL6, IL8, IL18 and IFN-γ. Mock n = 10, 10^6^ ffu HCV n = 5, 10^7^ ffu HCV n = 16.

To visualise the infiltration of human immune cells, liver sections were stained using an antibody specific against human CD45, a pan-leukocyte marker. Livers from mock-infected HIL mice showed evenly distributed human leukocytes with no obvious signs of inflammation (Fig 4A and 4B). At 20–28 weeks post infection, an increased amount of human CD45^+^ cells was observed in livers of HCV infected mice (Fig 4C–4F). In tumour regions, the accumulation of human leukocytes with patterns resembling granulomatous inflammation was observed within the tumours, especially at regions with steatosis (Fig 4D) and at the tumour peripheries (Fig 4E). Non-tumour regions in HCV infected livers also showed increased amount of infiltrating human leukocytes and granulomatous inflammation (Fig 4F).

In an earlier study, we have shown that HCV infection stimulated responses from human monocytes/macrophages and T cells during the early stage of infection (~9 weeks post infection) [25]. To investigate which immune cell types contributed to the chronic inflammation in this long term study, liver sections from mice 27 weeks post infection were stained with antibodies against human CD68, CD3 and CD20 which are markers of monocytes/macrophages, T cells and B cells respectively. CD68 staining revealed the presence of aggregated monocytes/macrophages in multiple granulomatous inflammation (Fig 4G). CD3^+^ T cells were also found infiltrated in various regions (Fig 4H), however, only a few CD20^+^ B cells were detected (Fig 4I). These findings are consistent with previous clinical observations that T cells and monocytes/macrophages both play a major role in HCV induced chronic hepatitis [33–37].

To analyse the human inflammatory cytokines in HCV infected HIL mice, plasma from HIL mice in all 3 cohorts were assayed for a panel of 9 inflammatory cytokines including interferon gamma (IFN-γ), monocyte chemoattractant protein 1 (MCP-1), interleukins (IL) 6, 8, 10, 12p70, 17A, 18 and 23. Of these cytokines, significantly increased levels of MCP-1, IFN-γ and IL18 were detected in plasmas of HIL mice infected with a 10^7^ ffu dose of HCV at 27–8 weeks post infection (Fig 4J). MCP-1, IFN-γ and IL18 were also reported to be elevated in the plasmas of HCV infected individuals and have been implicated in macrophage and T cell functions during HCV infection [38–41].

To determine if HCV infections had affected the immune profiles of HIL mice, absolute leukocyte counts coupled with flow cytometric analyses were performed on leukocytes collected from spleen and livers from HIL mice in cohorts 2 & 3 at 27–28 weeks post HCV infection. A representative flow cytometric gating strategy plot is shown as (S4 Fig).

**Fig 5:**
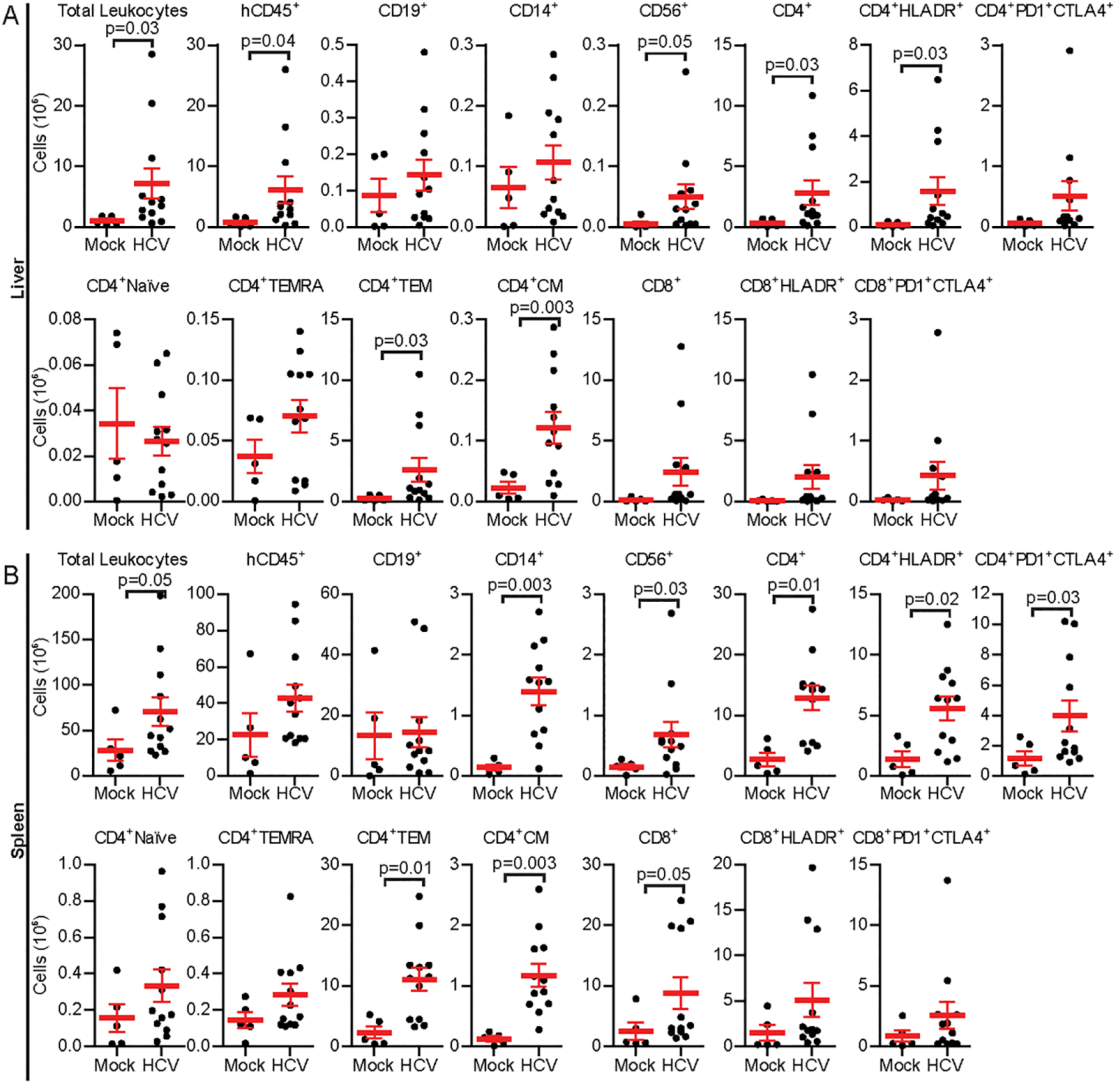
Human immune cell composition and phenotyping in HCV infected HIL mice. Leukocytes were prepared from livers and spleens of mock infected or mice infected with 10^7^ ffu of HCV 27 weeks post infection and analysed by flow cytometry. Shown are absolute numbers of various immune cell populations in (A) livers or (B) spleens of mock or HCV infected mice. Error bars represent standard error of the means. Mock n = 5, 10^7^ ffu HCV n = 12.

HIL mice infected with a dose of 10^7^ ffu of HCV had enlarged livers and spleens with increased numbers of leukocytes compared to mock controls (Liver, Mock: 1.09 ± 0.30 x 10^6^ cells, HCV: 7.19 ± 2.52 x 10^6^ cells, p = 0.03; Spleen, Mock: 36.51 ± 11.79 x 10^6^ cells, HCV: 70.64 ± 15.66 x 10^6^ cells, p = 0.05) (Fig 5A and 5B). Although no significant changes in proportions of different immune cell types were detected in the livers and spleens of HCV infected mice compared to mock infected mice (S5 Fig), analysis of liver leukocytes revealed significantly increased numbers of human CD45^+^ leukocytes, CD56^+^ NK cells and CD4^+^ T cells in HCV infected mice. Amongst the liver CD4^+^ T cells, CD4^+^HLADR^+^ activated T cells, CD4^+^CD45RA^-^CCR7^-^ effector memory and CD4^+^CD45RA^-^CCR7^+^ central memory cells were significantly increased compared to mock infected mice (Fig 5A). The spleens of HCV infected mice contained significantly increased numbers of human CD14^+^ monocyte/macrophages, CD56^+^ NK cells, total CD4^+^ T cells, CD4^+^HLADR^+^ activated T cells, CD4^+^PD1^+^CTLA4^+^ T cells, CD4^+^CD45RA^-^CCR7^-^ effector memory and CD4^+^CD45RA^-^CCR7^+^ central memory T cells, and total CD8^+^ T cells compared to mock infected mice (Fig 5B). Collectively these results are similar to clinical data obtained from chronically infected HCV patients [33, 40, 42–44].

### Localisation of exhausted like T cells and pro-tumour M2 macrophages in the livers of HCV infected HIL mice

**Fig 6:**
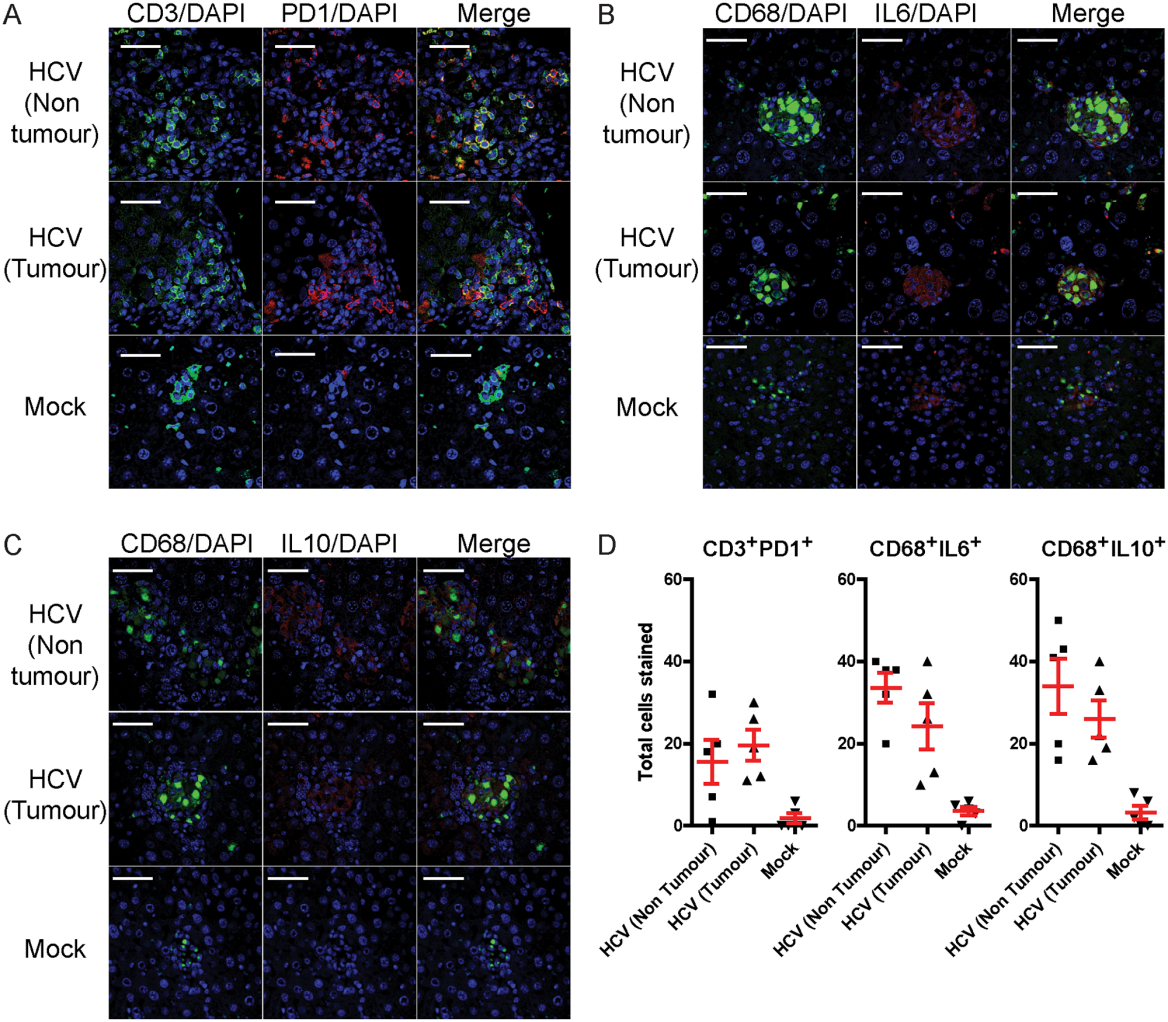
Deposition of human T cells and macrophages in non-tumour and tumour regions in the livers of HCV infected HIL mice. Livers sections from mock infected and HCV infected HIL mice 27 weeks post infection were stained for human CD3, PD1, CD68, IL6 and IL10. Representative images are shown for the stains of (A) CD3 and PD1, (B) CD68 and IL6 and (C) CD68 and IL10. Scale bars represent 50 μM. (D) Total number of CD3^+^PD1^+^, CD68^+^IL6^+^ or CD68^+^IL10^+^ cells were counted from five randomly selected 310 x 310 μm regions from mock liver sections, non-tumour or tumour regions from HCV infected HIL mice. Error bars represent standard error of the means.

One of the important dysfunctions that occur during chronic viral infections and tumorigenesis is the progressive exhaustion of T cells which express PD1 and CTLA4 [45, 46]. Although PD1^+^ T cells had been detected in HCV infected HIL mouse liver by flow cytometry (Fig 5A), immunofluorescence was also applied to visualise the location of these T cells within the liver. Histological analyses revealed that CD3^+^ T cells in livers from mock infected mice were generally dispersed evenly throughout the liver and were negative for PD1, whereas aggregation of CD3^+^PD1^+^ T cells were seen in inflamed regions in both non-tumour and tumour regions of HCV infected livers (Fig 6A).

Macrophages can be further classified into pro-inflammatory (M1) or pro-tumour (M2) phenotypes [47–49]. To visualise liver M1 or M2 macrophages, liver sections were stained using antibodies against CD68, a pan-macrophage marker with IL6 (Fig 6B) or IL10 (Fig 6C) which are the respective M1 or M2 macrophage markers. CD68^+^ macrophages within uninfected livers generally do not form multinucleated giant cell formations and expressed IL6 but not IL10, indicating that they are primarily of the M1 phenotype. In contrast, both M1 and M2 macrophages were seen within and outside of tumour regions in HCV infected livers, often forming multinucleated cell structures (Fig 6B and 6C). Quantification of PD1^+^ T cells, M1 and M2 macrophages revealed increased total numbers of all three cell types in the livers of HCV infected mice compared to mock infected mice (Fig 6D).

Collectively, our data suggests that HCV infections can trigger long-term inflammation and human immune cell infiltrations of livers in humanised mice. Most importantly, exhaustion and tumour associated phenotypes such as PD+ T cells and M2 macrophages, which are typically detected in patients with chronic hepatitis were reproduced in this model.

## Discussion

In our previous study, we described HCV mediated liver pathogenesis in HIL mice for up to 9 weeks post infection. While the onset of inflammation and liver fibrosis were noted, liver tumorigenesis was not detected potentially because a longer duration was required for the development of tumours. In this study, HCV infected HIL mice were analysed 20–28 weeks post infection and were found to have increased frequencies of liver abnormalities such as liver fibrosis, steatosis and granulomatous inflammation; symptoms also observed in patients with chronic HCV infections. These findings demonstrate that hepatitis C pathogenesis is able to progress beyond 27 weeks in the HIL mouse model. Despite the observations of chronic hepatitis, viraemia could not be detected from plasma or livers of infected mice. One of the limitations of this study is the difficulty of detecting HCV RNA using non-sacrificial sampling methods. We have demonstrated previously that HCV RNA could only be detected after extracting RNA from purified human hepatocytes in infected HIL mice [25], although this renders the liver tissue unusable for histopathological analyses. Here, although we were able to detect HCV RNA in fixed liver tissues 9 weeks post infection in RNA in situ hybridisation assays, no HCV RNA was detected at 20–28 weeks post infection. No HCV RNA could be detected either, from sera of infected mice at 9 or 20–28 weeks post infection. This suggests that initial infection of HCV was sufficient to induce onset of liver inflammation, which persist 28 weeks post infection potentially due to other factors such as secondary infections; an observation also seen in some HCV patients who have achieved SVR but without improvement in liver inflammation [50]. One of the observations supporting this hypothesis is the presence of granulomatous inflammation, shown to be mediated by CD68 expressing monocyte/macrophages, and is consistent with a chronic viral infection phenotype [51, 52]. This was accompanied by increased levels of MCP-1, a chemokine that functions by stimulating migration and infiltration of monocytes and macrophages (Fig 4J).

Significant human T cell responses, evidenced by increased numbers of effector memory, central memory and HLA-DR expressing CD4^+^ T cells were found in livers and spleens of HCV infected HIL mice. As shown in our previous work, the increased numbers of activated CD4^+^ T cells accounted for the elevated levels of plasma IFN-γ in infected HIL mice [25]. Although exhaustion of CD4^+^ and CD8^+^ T cells have been described by many clinical studies during chronic viral infection [44, 45], we found that there were more CD4^+^ T cells expressing PD1 and CTLA4 compared to CD8^+^ T cells in this model. One potential explanation for this is the observation that exhaustion of CD4^+^ T cells becomes apparent earlier during chronic viral infection as compared to CD8^+^ T cells [46]. In addition, another chronic inflammation associated phenotype is the accumulation of M2 Macrophages, which was also observed in this study. Collectively, our data showed that the immune cell types primarily responsible for sustaining chronic inflammation in infected HIL mice were human monocyte/macrophages and T cells. This is consistent with findings from the early stage of infection by HCV [25]. These observations support the rationale of specifically targeting the immune component to treat chronic HCV pathogenesis [53]. Even though mice in each cohort have received cells from the same foetal liver, variations of long term HCV pathogenesis were observed in these mice. This is likely explained by the differences such as the degree of immune cell and human hepatocyte reconstitution, environmental factors or other health status. Mouse models that support high levels of human hepatocyte reconstitution without a humanised immune system such as the SCID/Alb-uPA or Fah/Rag2/Il2rg mice have been shown recently to be capable of supporting long term HCV infection but fail to reproduce the pathological outcomes of hepatitis C infections [19, 20]. These observations along with our data and recent findings made in HCV infected AFC8-hu HSC/Hep mice [21] suggest that humanisations of both the immune system and liver are crucial for the progression of HCV mediated chronic liver inflammation.

Although the formation of liver tumours is a rare occurrence in this study, we report that HCV infected HIL mice can develop liver tumours by 20–28 weeks post infection with incidence rates of 2 of 31 (6%) HCV infected mice forming hepatic tumours. Clinical studies report that approximately 1–3% of chronically infected patients developed HCC which are diagnosed over a mean timeframe of 28±11 years [5, 6, 54]. Although none of the mice in this study developed chronic viral infections, our findings are similar in that 1 of 31 (3%) HCV infected mice developed HCC after 20–28 weeks post infection. The formation of liver tumours in HCV infected HIL mice without HCV viraemia suggest that this mouse model may mimic cases of clinical hepatitis C where patients previously infected with HCV still develop HCC after achieving SVR [55–58]. Since no long term viraemia could be detected at 20–28 weeks post infection, we hypothesise that initial infection by HCV contributes to the induction of chronic inflammation, which in turn unremittingly stimulates liver regeneration and may eventually lead to liver tumorigenesis. Majority of the mitotic cells within the tumours were human HSA^+^ cells, suggesting that this process had selectively transformed human hepatocytes. Since HIL mice possessed approximately 5% human hepatocyte reconstitution, it is possible that initial infections of a small percentage of hepatocytes could be sufficient to initiate tumorigenesis.

Further classification of the hepatocellular adenomas revealed that of 2 hepatocellular adenomas, 1 was inflammatory, 1 was HNF1-alpha inactivated and none were β-catenin mutated. Multiple adenomas formed in a single mouse were consistently of a single type. Clinical data estimates that the incidences of inflammatory, HNF1-alpha inactivated, β-catenin mutated and unclassified hepatocellular adenomas to be 50%, 35–40%, 10–15% and 10% respectively [59]. Hence, it is possible that only inflammatory and HNF1-alpha inactivated adenomas were detected in this study due to the small sample size. Our study of HCV infections in a novel humanised mouse model revealed that initial HCV infections of a small number of human hepatocytes may be sufficient to induce chronic liver inflammation, development of exhaustion immune cell phenotypes and selective transformation of human tumours in humanised mice. These findings provide new insight on HCV pathogenesis, human specific immune responses, and potentially provide a platform for drug testing against chronic hepatitis.

## Supporting information

S1 Table Details of mice cohorts used in this study. (TIF)

S2 Table Correlation of mice with elevated ALT and HSA levels throughout the 27 week course of study. (TIF)

S3 Table Serum ALT and HSA values. (TIF)

S1 Fig (A) RNA extracted from mouse sera or HCV spiked sera samples were converted to cDNA and analysed via qPCR. HCV RNA was detected to 23 copies per 100ul sera in the spiked samples. No HCV RNA was detected from mock or HCV infected sera at weeks 5, 9 or 28 post infection. (B) Detection of HCV RNA by fluorescence in situ hybridisation. Formalin fixed paraffin embedded liver sections from mock infected mice or HCV infected mice at 9 and 20 weeks were stained with anti-HSA antibodies (green) and RNA probe sets specific against HCV RNA (red). HCV RNA was detected in HSA expressing hepatocytes in the livers of HCV infected HIL mice at 9 weeks but not at 28 weeks post infection or in mock infected mice. Representative images are shown. Scale bars represent 20 μM.
(TIF)

S2 Fig Staining of liver sections with a HSA specific antibody showing background staining in normal mouse liver (A, D), in CpG oligodeoxynucleotide induced mouse liver tumours (B, E) or in HCV induced liver tumours in HIL mice (C, F). (A, B, D, E) Positive staining can be seen in the blood vessels and liver sinusoids but not in the mouse hepatocytes. (C, F) Positive staining of human hepatocytes within the hepatocellular adenoma and a portion of cells outside of the tumour demonstrates the specificity of the HSA antibody.
(TIF)

S3 Fig Classification of hepatocellular adenomas formed in HCV infected HIL mice. Liver sections containing hepatocellular adenomas were classified by staining with antibodies against β-catenin, glutamine synthetase and liver fatty acid binding protein as (A) HNF1α inactivated or (B) inflammatory hepatocellular adenomas.
(TIF)

S4 Fig Gating strategy for analysing the immune profiles of HIL mice. (TIF)

S5 Fig Immune profiles of HIL mice expressed as proportions of total human leukocytes. (TIF)
