# Supplementary figures and images for: Hepatitis C virus mediated chronic inflammation and tumorigenesis in the humanised immune system and liver mouse model
Source: PLoS One. 2017 Sep 8;12(9):e0184127. doi: 10.1371/journal.pone.0184127 (PMC5590885; doi:10.1371/journal.pone.0184127)

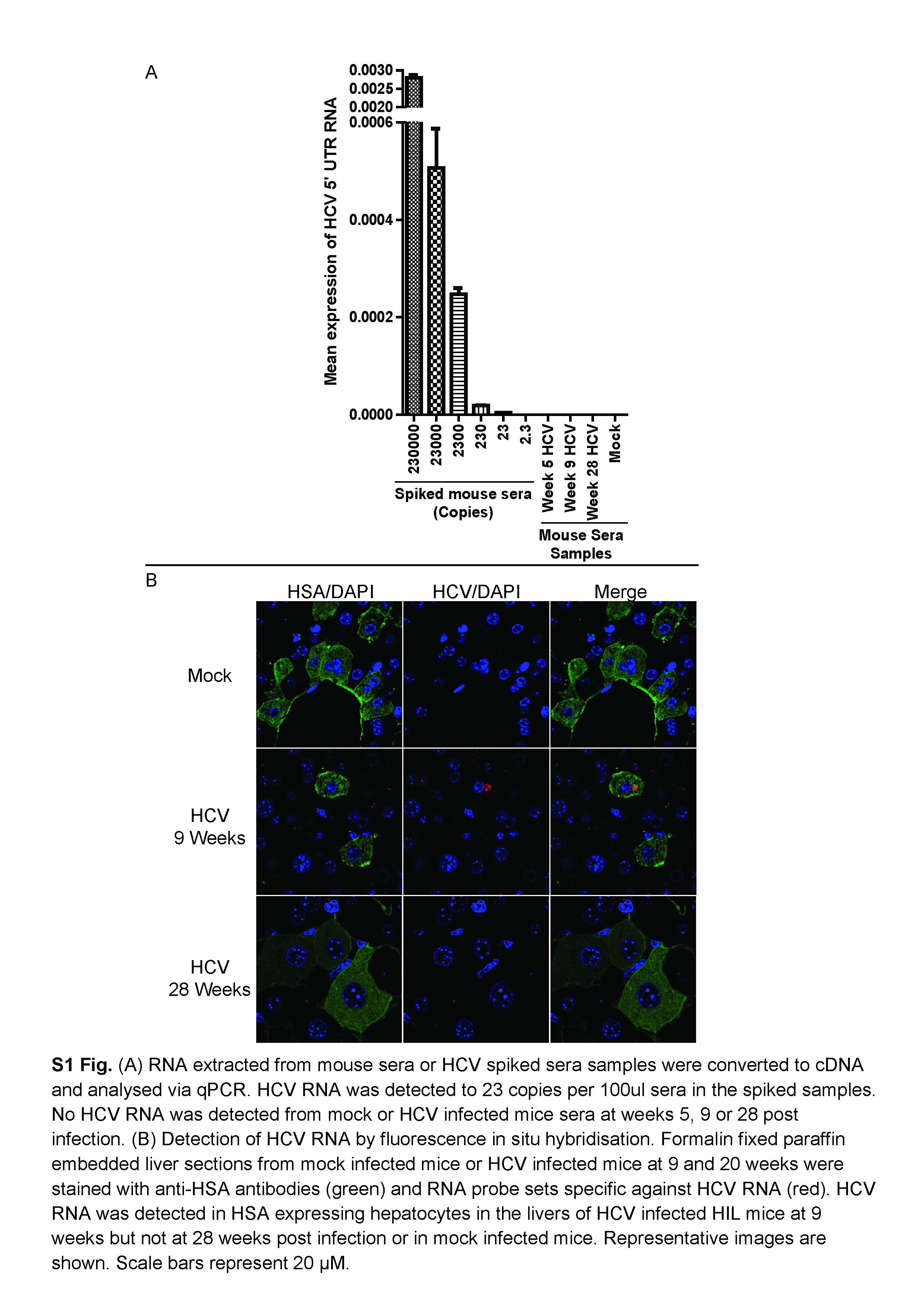

Supplement: S1 Fig — (A) RNA extracted from mouse sera or HCV spiked sera samples were converted to cDNA and analysed via qPCR. HCV RNA was detected to 23 copies per 100ul sera in the spiked samples. No HCV RNA was detected from mock or HCV infected sera at weeks 5, 9 or 28 post infection. (B) Detection of HCV RNA by fluorescence in situ hybridisation. Formalin fixed paraffin embedded liver sections from mock infected mice or HCV infected mice at 9 and 20 weeks were stained with anti-HSA antibodies (green) and RNA probe sets specific against HCV RNA (red). HCV RNA was detected in HSA expressing hepatocytes in the livers of HCV infected HIL mice at 9 weeks but not at 28 weeks post infection or in mock infected mice. Representative images are shown. Scale bars represent 20 μM. (TIF) [file pone.0184127.s004.tif]

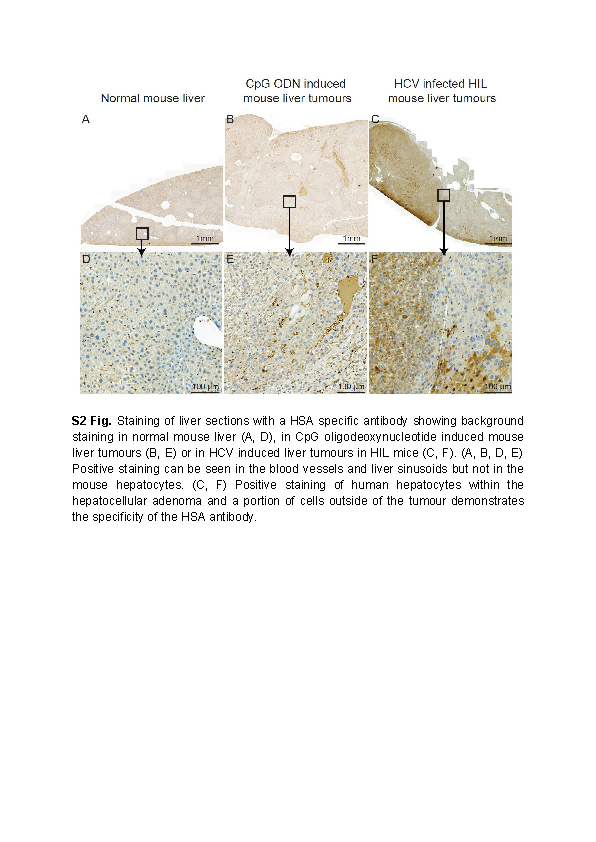

Supplement: S2 Fig — (A, B, D, E) Positive staining can be seen in the blood vessels and liver sinusoids but not in the mouse hepatocytes. (C, F) Positive staining of human hepatocytes within the hepatocellular adenoma and a portion of cells outside of the tumour demonstrates the specificity of the HSA antibody. (TIF) [file pone.0184127.s005.tif]

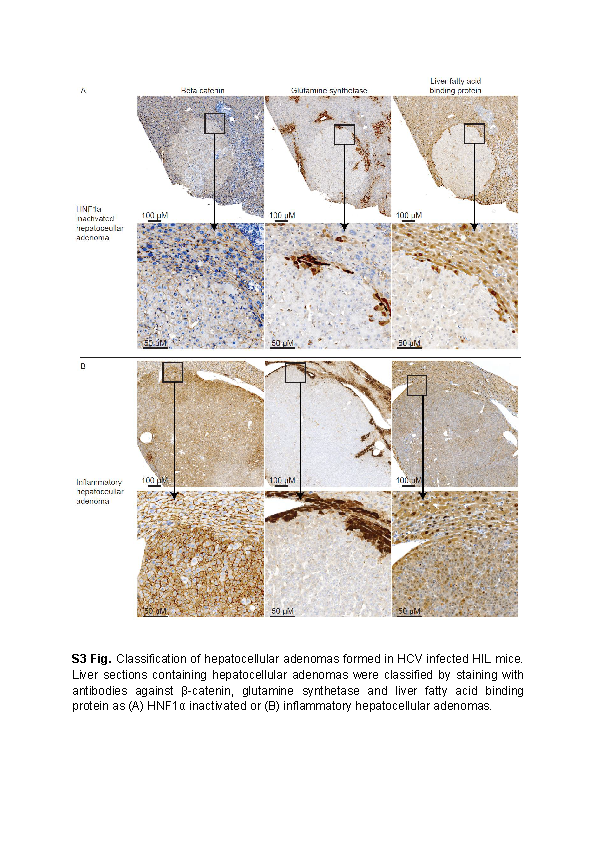

Supplement: S3 Fig — Liver sections containing hepatocellular adenomas were classified by staining with antibodies against β-catenin, glutamine synthetase and liver fatty acid binding protein as (A) HNF1α inactivated or (B) inflammatory hepatocellular adenomas. (TIF) [file pone.0184127.s006.tif]

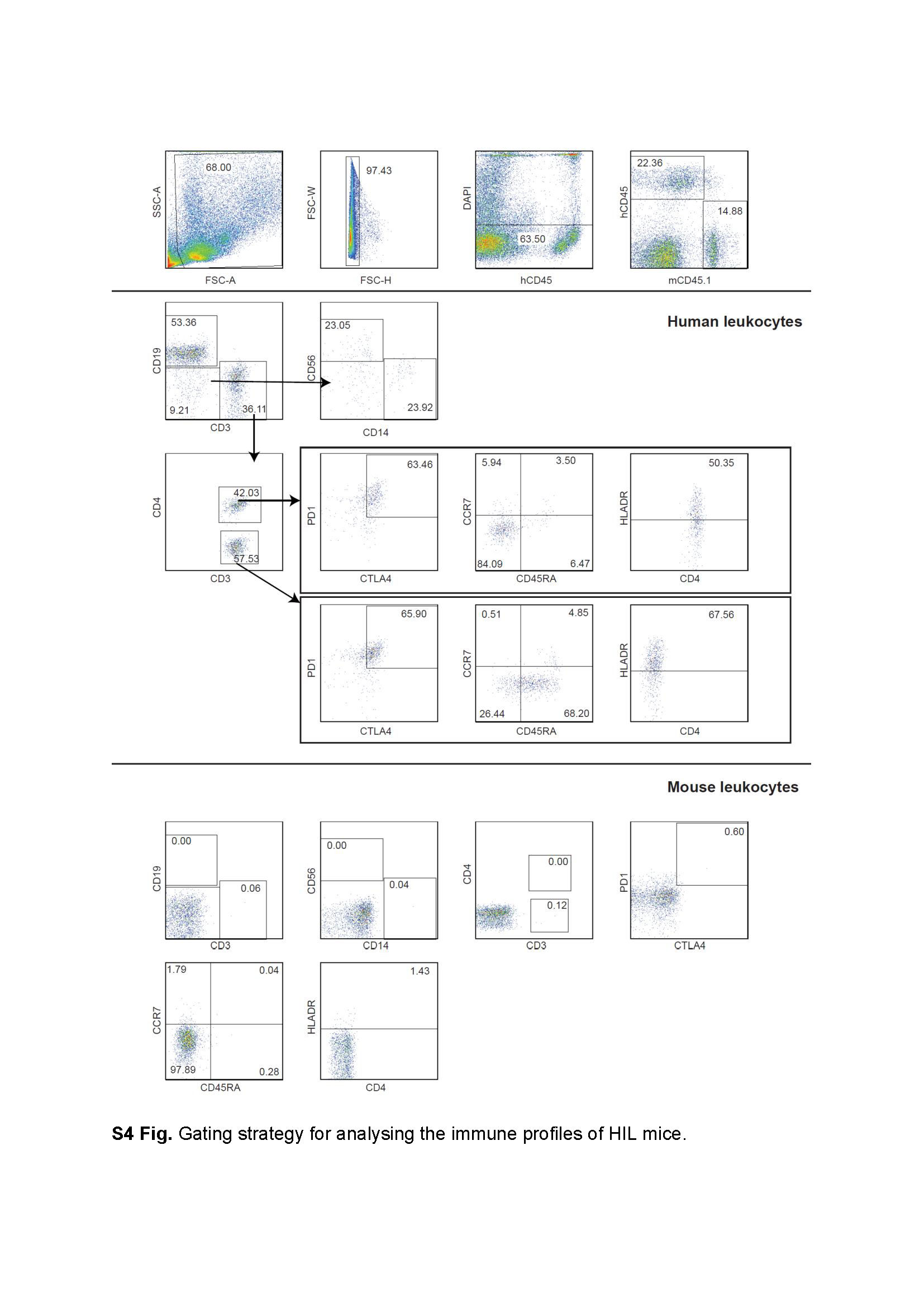

Supplement: S4 Fig — (TIF) [file pone.0184127.s007.tif]

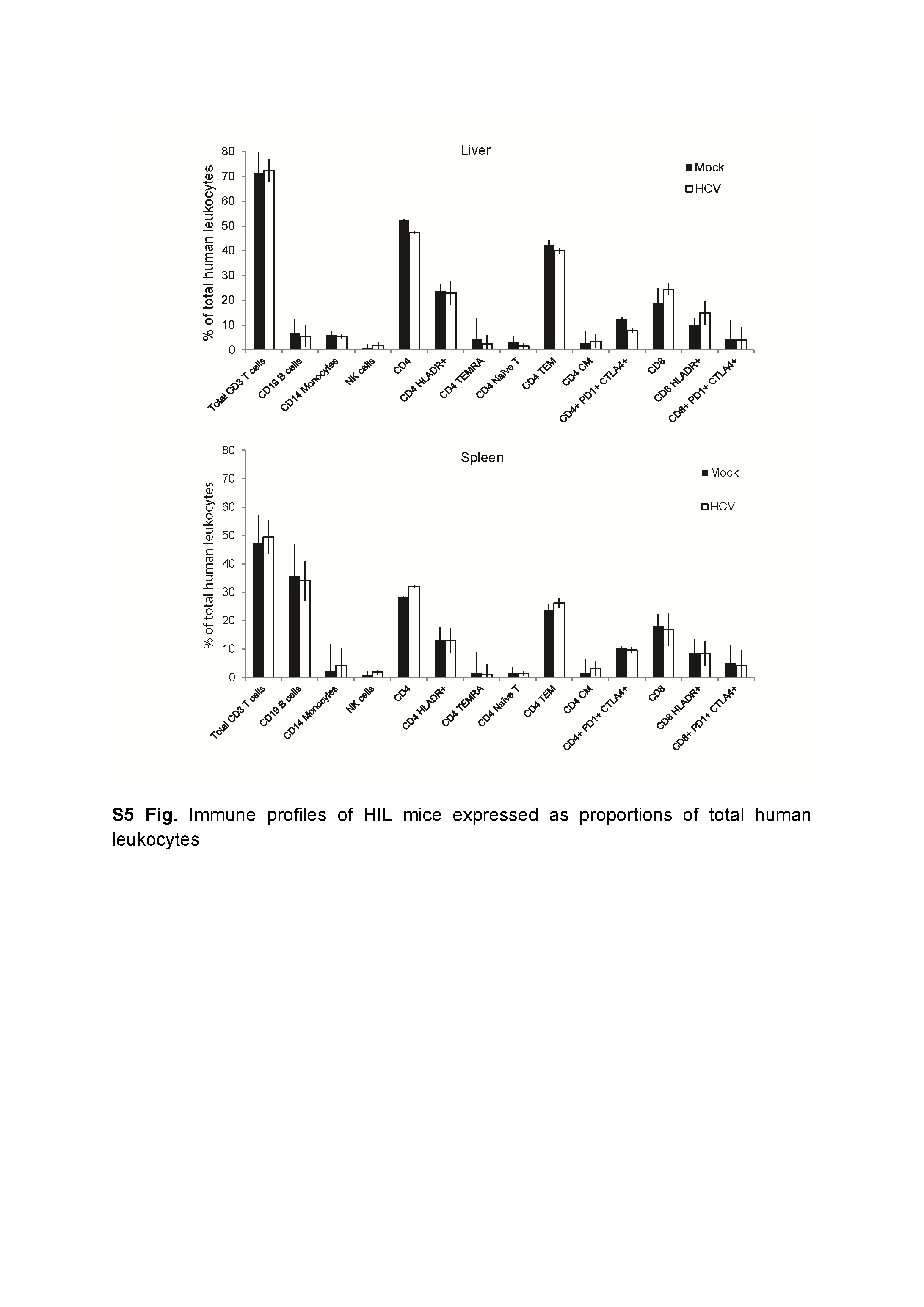

Supplement: S5 Fig — (TIF) [file pone.0184127.s008.tif]
